# Epidemiology of genital warts in the British population: implications for HPV vaccination programmes

**DOI:** 10.1136/sextrans-2018-053786

**Published:** 2019-02-05

**Authors:** Pam Sonnenberg, Clare Tanton, David Mesher, Eleanor King, Simon Beddows, Nigel Field, Catherine H Mercer, Kate Soldan, Anne M Johnson

**Affiliations:** 1 Mortimer Market Centre, Institute for Global Health, University College London, London, UK; 2 Department of Infectious Disease Epidemiology, London School of Hygiene & Tropical Medicine (LSHTM), London, UK; 3 Centre for Infectious Disease Surveillance and Control (CIDSC), Public Health England, London, UK; 4 Virus Reference Department, Public Health England, London, UK

**Keywords:** probability sample survey, genital warts, HPV

## Abstract

**Objectives:**

To estimate the prevalence of, and describe risk factors for, genital warts (GWs) in the British population, following the introduction of the bivalent (human papillomavirus (HPV)-16/18) vaccination programme in girls, and prior to the switch to quadrivalent (HPV-6/11/16/18) vaccine (offering direct protection against GWs) and compare this with GW diagnoses in the prevaccination era.

**Methods:**

Natsal-3, a probability sample survey in Britain, conducted in 2010–2012, interviewed 9902 men and women aged 16–44. Natsal-2, conducted in 1999–2001, surveyed 11 161 men and women aged 16–44. Both surveys collected data on sexual behaviour and sexually transmitted infection diagnoses using computer-assisted interview methods.

**Results:**

In Natsal-3, 3.8% and 4.6% of sexually experienced men and women reported ever having a diagnosis of GWs, with 1.3% of men and 1.7% of woman reporting a GWs diagnosis in the past 5 years. GWs were strongly associated with increasing partner numbers and condomless sex. Diagnoses were more frequent in men who have sex with men (MSM) (11.6% ever, 3.3% past 5 years) and in women reporting sex with women (10.8% ever, 3.6% past 5 years). In the age group who were eligible for vaccination at the time of Natsal-3 (16–20 years), a similar proportion of same-aged women reported a history of GWs in Natsal-2 (1.9%, 1.1–3.4) and Natsal-3 (2.6%, 1.5–4.4).

**Conclusions:**

These data provide essential parameters for mathematical models that inform cost-effectiveness analyses of HPV vaccination programmes. There was no evidence of population protection against GWs conferred by the bivalent vaccine. Even with vaccination of adolescent boys, vaccination should be offered to MSM attending sexual health clinics.

## Introduction

Genital warts (GWs) are the most common viral sexually transmitted infection (STI) diagnosed among genitourinary medicine clinic (GUM) attendees in the UK^1^ and globally.^2^ The diagnosis and treatment of GWs also has associated individual and healthcare costs: in 2008, the annual expenditure on GWs in England was estimated at £16.8 million,^3^ and each episode is estimated to have a QALY loss equivalent to 6.6 days of healthy life lost.^4^

In 2008, the UK began a human papillomavirus (HPV) immunisation programme in adolescent girls (aged 11–12 years, with catch-up to 17 years), initially using the bivalent vaccine which protects against HPV types 16 and 18, the most frequent causes of cervical cancer. In 2012, the programme switched to quadrivalent HPV vaccine which additionally protects against HPV types 6 and 11, which are responsible for over 90% of cases of GWs.^5^

A recent systematic review^6^ of the effectiveness of the quadrivalent vaccine, following 10 years of real-world experience, reported that, as early as 2 years after starting a vaccination programme in girls, and with high coverage such as in Australia and Denmark, there was a rapid and marked reduction in the incidence of GWs among women eligible for vaccination and some benefit conferred to similar-aged heterosexual men.^7^ Reductions in GWs in men who have sex with men (MSM) have been, as expected, less evident.^8^ In England, surveillance data from GUM clinics have shown an unexpected reduction in diagnoses of GWs between 2009 and 2011,^9^ particularly in women in the age groups offered bivalent vaccination and, to a lesser extent, in similar-aged heterosexual men, and this has continued to 2014.^10^ This ecological observation, together with findings of moderate efficacy against some low-risk HPV types in a posthoc analysis of the PATRICIA (Papilloma TRIal against Cancer In young Adults) trial of the bivalent vaccine,^11^ has led to a hypothesis that the bivalent vaccine may confer a modest cross-protective effect against GWs.

The availability of population-based comparison data from the prevaccination era is important to demonstrate population impact.^6^ Furthermore, it is important to monitor the prevalence of GWs in different subgroups, to ensure that vaccination programmes are widely accessed, reach those at greatest risk and reduce health inequalities. For Britain, the National Surveys of Sexual Attitudes and Lifestyles (Natsal) are able to capture the population burden of STIs and link this to detailed behavioural information. This includes people who access services for testing, diagnosis and treatment including, but not limited to sexual health (GUM) clinics, which are the main contributors to STI surveillance. The timings of the Natsal surveys allow estimation of the population effects of HPV vaccination: Natsal-2 was conducted in 1999–2001 prior to the introduction of any HPV vaccination, with Natsal-3 in 2010–2012 taking place after the introduction of bivalent vaccination (but prior to the switch to quadrivalent vaccine).

This paper reports the prevalence of reported diagnoses of, and risk factors for, GWs in the British population in Natsal-3. We examine changes in reported prevalence of GW diagnoses between the birth cohorts eligible for vaccination in Natsal-3, with those of equivalent age in the prevaccination era in Natsal-2, to assess whether there is population-based evidence of cross-protection against GWs.

## Materials and methods

### Participants and procedures

Natsal-3 interviewed 15 162 men and women aged 16–74 in 2010-2012. Details of the study methods have been described previously.^12^ Briefly, we used a multistage, clustered, stratified probability sample design. The response rate was 57.7%. Participants were interviewed through a combination of face-to-face computer-assisted personal interview and computer-assisted self-interview for the more sensitive questions, including on their experience of STI diagnoses. Participants were asked if they had ever been told by a doctor or other healthcare professional that they had any of a list of different STIs, including GWs, and if so, when they were last diagnosed with GWs. Natsal-2 (undertaken in 1999–2001 surveyed 11 161 men and women aged 16–44, with a response rate of 65.4%.^13^ The surveys use similar methodology to allow comparison over time. HPV catch-up vaccination coverage in Natsal-3 participants was 61.5%.^14^ Vaccine uptake varied by school year at eligibility, with 72.9% of women eligible at 14 years reporting having received all three doses, compared with only 50.6% of women eligible at 17 years.^15^

### Statistical analysis

Of the 3809 men and 5510 women aged 16–44 years in Natsal-3, we included sexually experienced participants with information on GW history (3570 men (93.7%) and 5257 women (95.4%)) in the main analysis. Analysis accounts for the stratification, clustering and weighting of the sample. We describe the prevalence of reported history of GWs over different time periods by age. In those reporting at least one partner in the past 5 years (of either gender), we stratify this according to experience of same-sex sex during this timeframe. Age-adjusted odds ratios (aORs) are calculated for the association between key behavioural risk factors and a history of GWs in the past 5 years. Data for comparison between Natsal-2 (4267 men and 5869 women) and Natsal-3 were available for sexually experienced men and women aged 16–44 years. We describe the prevalence of reported history of GWs in the age group of the birth cohorts in Natsal-3 who would have been eligible for vaccination (aged 16–20 years) and present a prevalence ratio with 95% CIs. Data were analysed using Stata (V.14.1).

### Ethics

Natsal-3 was granted ethical approval by the Oxfordshire Research Ethics Committee A (Reference: 09/H0604/27). Natsal-2 obtained ethical approval from University College Hospital, North Thames Multicentre and all local research ethics committees in Britain.

## Results

Using data from Natsal-3 (2010–2012), table 1 shows how the proportion of the population reporting a diagnosis of GWs varied by age and gender. Overall, 3.8% of sexually experienced men and 4.6% of sexually experienced women aged 16–44 had ever had a diagnosis of GWs. Ever diagnosis increased with age, plateauing at around 5% at age 25 in men and age 20 in women. In total, 1.3% of men and 1.7% of women had a diagnosis in the past 5 years, with 0.3% of men and women reporting a diagnosis in the past year.

**Table 1:** Reported diagnoses of genital warts, ever, in the past 5 years and in the past year, in British men and women aged 16–44*

	16–19		20–24		25–34		35–44		Total aged 16–44	
	%	95% CI	%	95% CI	%	95% CI	%	95% CI	%	95% CI
**Men**										
Denom. (unwt, wt)†	578, 370		785, 620		1432, 1284		775, 1367		3570, 3642	
Genital warts										
Ever	0.2%	(<0.1 to 0.9)	1.8%	(1.0 to 3.3)	4.7%	(3.6 to 6.1)	4.9%	(3.4 to 6.8)	3.8%	(3.0 to 4.7)
Past 5 years	0.2%	(<0.1 to 0.9)	1.8%	(1.0 to 3.3)	2.0%	(1.3 to 3.0)	0.6%	(0.3 to 1.5)	1.3%	(0.9 to 1.7)
Past year	0.2%	(<0.1 to 0.9)	0.4%	(0.1 to 1.4)	0.4%	(0.1 to 1.1)	0.2%	(<0.1 to 1.3)	0.3%	(0.2 to 0.6)
**Women**										
Denom. (unwt, wt)†	667, 341		1059, 621		2362, 1304		1169, 1397		5257, 3662	
Genital warts										
Ever	1.5%	(0.7 to 2.9)	5.0%	(3.6 to 7.0)	4.8%	(3.9 to 6.0)	4.9%	(3.8 to 6.4)	4.6%	(4.0 to 5.3)
Past 5 years	1.1%	(0.6 to 2.3)	4.3%	(2.9 to 6.3)	1.6%	(1.1 to 2.4)	0.6%	(0.3 to 1.3)	1.7%	(1.3 to 2.1)
Past year	0.7%	(0.3 to 1.7)	1.2%	(0.6 to 2.5)	0.1%	(<0.1 to 0.6)	<0.1%	(<0.1 to 0.4)	0.3%	(0.2 to 0.6)

*Data from Natsal-3 2010–2012.

†Denominators are those with 1+partner ever.

Diagnoses were more frequent in MSM (11.6% ever and 3.3% past 5 years) than men who reported exclusively having sex with women (3.6% ever and 1.2% past 5 years) (table 2). Similarly, diagnoses were more frequent in women reporting sex with women (WSW) (10.8% ever and 3.6% past 5 years) than in women who reported exclusively having sex with men (4.3% and 1.6%, respectively).

**Table 2:** Reported diagnoses of genital warts in those aged 16–44 years by same-sex behaviour in the past 5 years*

	Men				Women			
	MSEW†		MSM†		WSEM†		WSW†	
	%	95% CI	%	95% CI	%	95% CI	%	95% CI
Denom. (unwt, wt)‡	3377, 3471		137, 116		4881, 3430		304, 189	
Genital warts								
Ever	3.6%	(2.9–4.4)	11.6%	(5.7–22.1)	4.3%	(3.6–5.0)	10.8%	(7.2–15.8)
Past 5 years	1.2%	(0.9–1.7)	3.3%	(1.2–8.3)	1.6%	(1.2–2.0)	3.6%	(2.0–6.5)
Past 1 year	0.3%	(0.1–0.6)	0.4%	(0.0–2.5)	0.3%	(0.2–0.6)	0.7%	(0.2–2.6)

*Data from Natsal-3 2010–2012.

†MSEW (men who have sex exclusively with women); MSM (men who have sex with men, including men who have sex with men and women).

‡Denominators are those with 1+partner in the past 5 years.

WSEM, women who have sex exclusively with men; WSW, women who have sex with women, including women who have sex with men and women.

**Figure 1:**
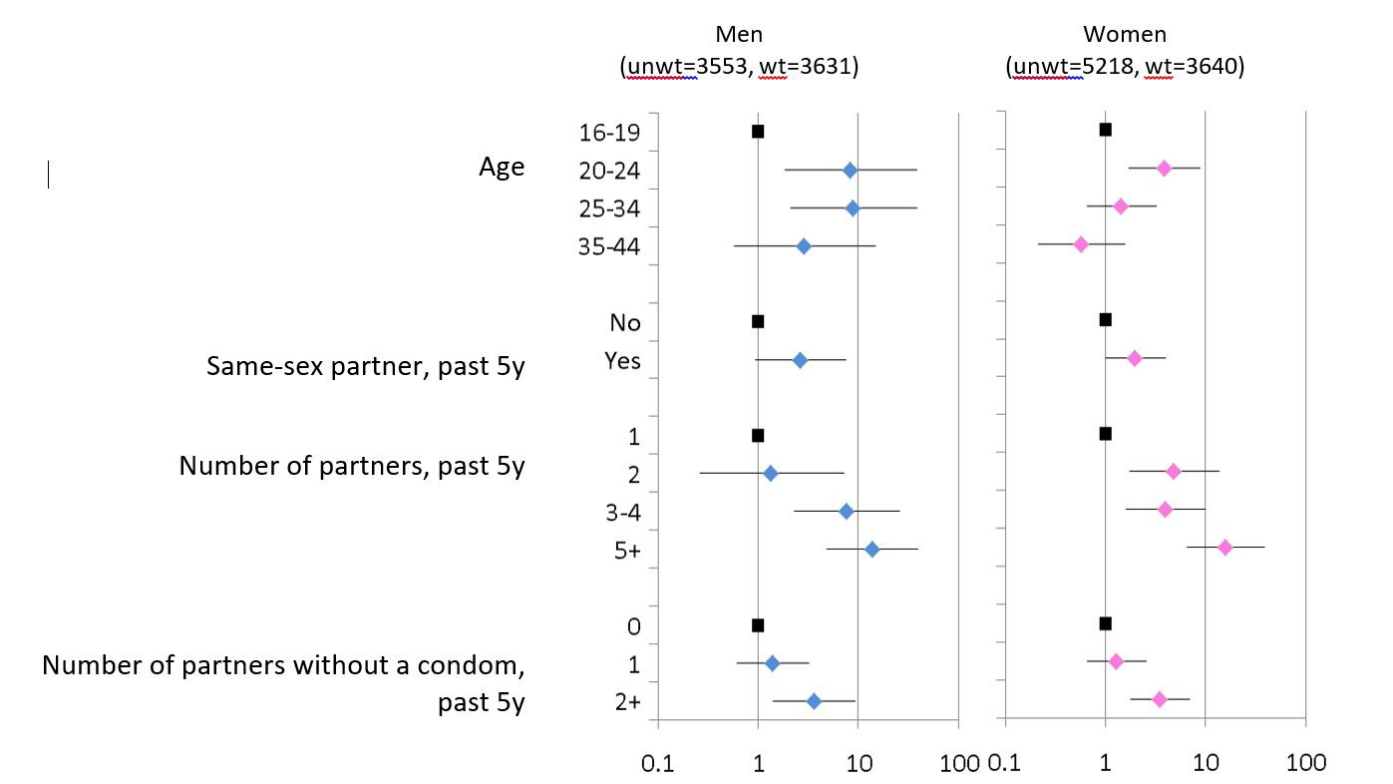
Factors associated with a diagnosis of genital warts in the past 5 years, in men and women aged 16–44.*See separate jpg file. *Data from Natsal-3. denominators are those with 1+partner in the past 5 years. Age-adjusted ORs and 95% CIs.

A diagnosis of GWs was associated with age, but there were no significant associations with ethnicity, area-level deprivation or educational status (data not shown). Figure 1 shows behavioural factors associated with a diagnosis of GWs in the past 5 years in those with one or more partner in this timeframe. For both men and women, a warts diagnosis was strongly associated with increasing partner numbers and condomless sex.

Those reporting a same-sex partner in the past 5 years were also more likely to report a history of GWs (aOR 2.66 (0.94–7.50) in men and 1.99 (1.00–3.94) in women); however, this effect was no longer significant after adjusting further for partner numbers (adjusted OR 0.95 (0.29–3.13) in men and 0.92 (0.46–1.84) in women).

In Natsal-2, data were only available on a history of GWs ever. This was reported in 3.7% (3.1%–4.3%) of men and 4.2% (3.7%–4.8%) of women aged 16–44 years, very similar proportions to those reported a decade later in Natsal-3. Specifically, in women in the birth cohorts who would have been eligible for vaccination in Natsal-3 (16–20 years at time of interview), a similar proportion of same-aged women reported a history of GWs in Natsal-2 (13/556, weighted prevalence 1.9%, 1.1–3.4) and Natsal-3 (21/870, weighted prevalence 2.6%, 1.5–4.4), with a prevalence ratio, comparing Natsal-3 to Natsal-2, of 1.35 (95% CI: 0.61 to 2.98).

## Discussion

Repeated cross-sectional population-based decennial surveys, such as Natsal, can provide baseline and postintervention estimates of prevalence, behaviour and uptake of services for national programmes. These are vital to monitor impact, model cost-effectiveness and guide decision-making on interventions, including vaccination strategies. Natsal data complements surveillance but is also able to provide important parameters for modelling HPV transmission dynamics that cannot be ascertained from clinic-based data, such as lifetime GW diagnoses and detailed sexual behaviour.

A diagnosis of GW was associated with markers of more risky sexual behaviours, similar to those for other STIs, including high-risk HPV infection.^14^ GWs were less common in heterosexual men and women in the general population, compared with men and women reporting same-sex behaviour. During the same time period as Natsal-3, a cross-sectional survey of ~500 MSM attending a London GUM clinic was undertaken.^16^ In this clinic-attending population of MSM, nearly one-third had a previous diagnosis of GWs ever (30.3%), 1 in 10 had a GWs diagnosis in the past year (9.8%) and in many cases they had had more than one episode.^17^ Our finding is consistent with knowledge that MSM have a higher incidence of HPV infection and related disease.^18^ This knowledge, along with the expectation that MSM will benefit less from herd protection from the vaccination of women, informed the Joint Committee on Vaccination and Immunisation’s (JCVI) advice in 2016 that quadrivalent vaccination should be offered to MSM attending sexual health and HIV clinics, as a cost-effective intervention.^19^ Following this advice, a pilot of HPV vaccination for MSM attending sexual health and HIV clinics across England was introduced in summer 2016, with similar vaccination programmes introduced nationally in Wales (since April 2017)^20^ and Scotland (since July 2017).^21^ The pilot showed feasibility and acceptability (45.5% recorded first dose uptake) and a national HPV vaccination programme for MSM (up to and including 45 years of age) is being rolled-out from April 2018.^22^ The recommendation to offer vaccination up to this age is based on the knowledge that while the vaccine may be more effective in those without previous HPV exposure (who are likely to be younger), the majority of MSM attending clinics have not been infected with all HPV-types in the vaccine^16^ and high-risk sexual behaviour, which is not restricted to young MSM, may result in repeated exposure.

Of note is that the prevalence estimates of ever having a diagnosis of GWs in MSM and WSW in the general population were similar at ~11%. The numbers of women who have sex exclusively with women in Natsal are very small, and the higher prevalence in WSW is likely to be in women having sex with both men and women. WSW have increased risk of STIs, higher partner numbers and other high-risk behaviours^23^ but the higher odds of GWs in WSW was no longer significant after adjusting for partner numbers. Nevertheless, given the high prevalence of GWs as well as lower uptake of cervical screening,^15^ it is important that WSW are included in health promotion messages and that vaccination coverage is high in this group. Women reporting more than five partners were at even higher risk (figure 1). This supports mop-up vaccination in these women if they attend sexual health services while in the eligible age group, if they had missed one or more doses in the routine programme.

Using data from Natsal-2 and Natsal-3, we have previously shown an early indication of population-based effectiveness of the bivalent HPV vaccine in women in the age groups eligible for vaccination, with a ~50% reduction seen in the prevalence of HPV-16/18 in 18–20 year olds, but no observed change in prevalences of cross-protective types or in women aged 21–44.^14 24^ We have also previously reported that the prevalence of HPV-6/11 in urine in women in the age group eligible for vaccination had not reduced significantly (9.5% in Natsal-2 vs 8.9% in Natsal-3, an age-adjusted prevalence ratio of 0.91 (0.44–1.89)).^24 25^ Likewise, in this paper, we found no change in the reporting of GW diagnoses following introduction of bivalent vaccination.

Our findings concur with the lack of protection against GWs observed in bivalent vaccine-immunised women in a number of studies^26–28^ and with the finding of no reduction in HPV-6 or HPV-11 infection in England^29^ or Scotland.^30^ Together, these data provide some evidence to refute the hypothesis of the HPV-16/18 vaccine conferring a cross-protective effect against HPV-6/11 and GWs. It is, however, possible that the timing of Natsal-3 relative to the start of the vaccination programme means that it was too soon to detect an effect on GWs and low risk HPV types, particularly since catch-up uptake was ~60% (but early enough to ascertain the greater direct effect on HPV-16/18).^14^ Small numbers may also have resulted in insufficient power to detect a significant difference in the prevalence ratio. Reductions in GWs were seen in the surveillance data by 2011: possible reasons for this should have generated the same effects in Natsal-3, with the exception of changes in service use or other artefacts and measurement errors (when looking at diagnosis trends) in the surveillance data.

This paper presents data from Natsal-3, prior to the switch to quadrivalent vaccine. Based on data from other countries and that in the UK, there has already been high coverage of quadrivalent vaccine in girls for 5 years, large reductions in population HPV-6/11 prevalence, and in diagnoses of GWs in both women and men, are already expected. Indeed, this is the case: in 2017, the rate of first episode GWs diagnoses among females aged 15–17 attending specialist sexual health services was 89% lower compared with 2009, with a decline of 70% in same aged heterosexual males over this time period, suggesting substantial herd protection.^31^ The impact of selective vaccination of MSM attending GUM/HIV clinics is expected to become evident in due course, as the vaccination programme started more recently and uptake is opportunistic and so will likely accrue more gradually. Following the recent JCVI recommendation to extend the adolescent girls programme to boys,^32^ even further reductions, in a shorter timeframe, are to be expected. As discussed above, even with a vaccination programme that includes adolescent boys, there remains a case for continuing to offer vaccination to MSM attending sexual health clinics up to age 45.^19^ In addition to the high burden of infection and disease, a large proportion of MSM attending clinics may not have been born in the UK (>50% in the London study),^16^ including those from countries without a vaccination programme.

Cost-effectiveness analyses will continue to be needed to inform the UK HPV immunisation programme, for example, as new vaccines enter the market. Updating and extending this evidence is therefore helpful to reduce error and uncertainty within such analyses. These data may also be informative for other service and treatment planning, for example in countries considering introduction of, or changes to, a HPV vaccination programme. Further real-world evaluation of the impact of HPV vaccination on GWs should include monitoring in sexual health clinics, as part of routine surveillance and in stand-alone research studies, complemented by population-based surveys.

**Key messages**

- In the British population, 3.8% of sexually experienced men and 4.6% of sexually experienced women aged 16–44 reported ever having a diagnosis of genital warts (GWs).
- GWs were more common in men and women reporting same-sex behaviour (11% ever diagnosed with GWs).
- There was no evidence of the bivalent vaccine conferring a cross-protective effect against GWs.
- Cost-effectiveness analyses, and real-world evaluation, will continue to be needed to inform UK human papillomavirus (HPV) immunisation policy, as changes are made to the vaccination programme (eg, vaccinating boys) and new vaccines enter the market (eg, nonavalent HPV vaccine).
